# Predicting rectal cancer prognosis from histopathological images and clinical information using multi-modal deep learning

**DOI:** 10.3389/fonc.2024.1353446

**Published:** 2024-04-15

**Authors:** Yixin Xu, Jiedong Guo, Na Yang, Can Zhu, Tianlei Zheng, Weiguo Zhao, Jia Liu, Jun Song

**Affiliations:** ^1^ Department of General Surgery, The Affiliated Hospital of Xuzhou Medical University, Xuzhou, Jiangsu, China; ^2^ Artificial Intelligence Unit, Department of Medical Equipment Management, Affiliated Hospital of Xuzhou Medical University, Xuzhou, China; ^3^ Institute of Digestive Diseases, Xuzhou Medical University, Xuzhou, Jiangsu, China

**Keywords:** rectal cancer, survival prediction, deep learning, multi-modal data, machine learning

## Abstract

**Objective:**

The objective of this study was to provide a multi-modal deep learning framework for forecasting the survival of rectal cancer patients by utilizing both digital pathological images data and non-imaging clinical data.

**Materials and methods:**

The research included patients diagnosed with rectal cancer by pathological confirmation from January 2015 to December 2016. Patients were allocated to training and testing sets in a randomized manner, with a ratio of 4:1. The tissue microarrays (TMAs) and clinical indicators were obtained. Subsequently, we selected distinct deep learning models to individually forecast patient survival. We conducted a scanning procedure on the TMAs in order to transform them into digital pathology pictures. Additionally, we performed pre-processing on the clinical data of the patients. Subsequently, we selected distinct deep learning algorithms to conduct survival prediction analysis using patients’ pathological images and clinical data, respectively.

**Results:**

A total of 292 patients with rectal cancer were randomly allocated into two groups: a training set consisting of 234 cases, and a testing set consisting of 58 instances. Initially, we make direct predictions about the survival status by using pre-processed Hematoxylin and Eosin (H&E) pathological images of rectal cancer. We utilized the ResNest model to extract data from histopathological images of patients, resulting in a survival status prediction with an AUC (Area Under the Curve) of 0.797. Furthermore, we employ a multi-head attention fusion (MHAF) model to combine image features and clinical features in order to accurately forecast the survival rate of rectal cancer patients. The findings of our experiment show that the multi-modal structure works better than directly predicting from histopathological images. It achieves an AUC of 0.837 in predicting overall survival (OS).

**Conclusions:**

Our study highlights the potential of multi-modal deep learning models in predicting survival status from histopathological images and clinical information, thus offering valuable insights for clinical applications.

## 1 Introduction

Colorectal cancer (CRC) is a malignant tumor that presents a substantial risk to human well-being. Global figures indicate that the number of newly diagnosed cases of colorectal cancer in 2020 exceeded 1.9 million, resulting in 940,000 deaths. This places colorectal cancer as the third most common disease in terms of incidence and the second leading cause of death (1). Rectal cancer patients account for approximately 57.9% of colorectal cancer patients in China (2). Rectal cancer has a significantly greater likelihood of local pelvic recurrence compared to colon cancer, and individuals who experience pelvic recurrence have a worse prognosis (3). Stratifying rectal cancer patients is crucial for developing more precise and tailored treatment based on their clinical features (4). The tumor-node-metastasis (TNM) staging system, which relies on histological examination, has been widely regarded as the fundamental tool for predicting prognosis and determining treatment decisions in colorectal cancer (CRC) for many years (5, 6). Nevertheless, it remains inadequate for accurately forecasting the fate of certain rectal patients, specifically those in clinical stages II and III (7, 8). The workload in a typical pathology laboratory is substantial because of the high prevalence of rectal cancer. Additionally, the inclusion of an expanding array of morpho-molecular characteristics to be analyzed and documented has made the prognosis a laborious and time-consuming task (9). Hence, it underscores the imperative to discover supplementary prognostic and/or predictive biomarkers that go beyond the existing staging system, in order to enhance prognosis and therapeutic decision-making.

Deep learning is a machine learning methodology that use numerous layers of processing and connections to acquire knowledge about intricate relationships between input data and intended output, drawing on a vast collection of labeled instances (10). Deep learning is a method that works with raw data and autonomously constructs its own representations for recognizing patterns, hence removing the requirement to explicitly define rules or features (11). In recent times, deep learning methods have demonstrated impressive results in several medical image analysis applications. Echle et al. conducted a study on 8836 cases of all stages of colorectal cancer (CRC) in order to create a model that can identify microsatellite instability (MSI) cancers using pathological pictures (12). Kiehl et al. showed that Lymph Node Metastasis (LNM) could be predicted by DL models with a good performance (13). Nevertheless, the potential of combining H&E images with other data sources, such as clinical information, to predict prognosis for rectal cancer has not yet been investigated.

Multi-modal analyses involve the examination of information from several sensory modalities that have distinct ways of creation and internal structures. This strategy entails examining the associations between disparate datasets for each modality. The objective of this work is to utilize multi-modal deep learning as the first step in developing a prognostic prediction model using clinical data and digital images of hematoxylin and eosin (H&E)-stained tissue microarrays (TMAs) collected from rectal cancer patients. The primary objective is to provide efficient, non-intrusive, and precise prognosis forecasting and personalized therapy for individuals with rectal cancer. This work aims to offer novel insights into precision medical research on rectal cancer.

## 2 Materials and methods

### 2.1 Dataset preparation

#### 2.1.1 Clinical data

The study included patients diagnosed with rectal cancer by pathological examination and meeting the selection criteria from January 2015 to December 2016. The clinical data of these patients were gathered from the electronic medical system. The criteria for inclusion were as follows: (1) The patient was diagnosed with rectal cancer by pathological examination. We had access to comprehensive clinical data, pathological data, and surgical follow-up information. Patients were eliminated according to the following criteria: (1) Preoperative neoadjuvant therapy, such as chemotherapy, radiotherapy, chemoradiotherapy, immunotherapy, molecular targeted therapy, etc.; (2) History of abdominal malignancies or inflammatory diseases; (3) Currently having an unresectable primary tumor lesion or distant metastases (such as in the liver or peritoneum) discovered during surgical exploration.

The baseline information, including age at diagnosis, sex, tumor size, Lauren type, depth of invasion (T stage), lymph node metastasis (N stage), distant metastasis (M stage), TNM stage, Ki-67 levels, P53 mutation status, Red blood cells (RBC)count, White blood cells(WBC)count, lymphocyte count, albumin level, platelet count, hemoglobin (HB) level, ALT level, AST level, albumin level, creatinine (Cr) level, Urea level, carcinoembryonic antigen (CEA) level, and carbohydrate antigen 199 (CA199) level, were obtained from the electronic medical system. The TNM stage was reclassified according to the eighth edition of the AJCC Cancer Staging Manual of the American Joint Committee on Cancer.

The main results focused on the 5-year overall survival (OS). The patients underwent regular follow-up assessments at intervals of 3 months throughout the initial 2-year postoperative period, every 6 months for the subsequent 3 years, and annually afterward. The period of follow-up was measured from the moment of surgery until the most recent follow-up, and the survival status at that time was recorded. The term “overall survival” refers to the period of time between a surgical procedure and the occurrence of death due to any cause. When dealing with clinical data, our initial step is to encode all discrete data and subsequently normalize them based on the distribution of continuous data. The primary methods employed for data augmentation are random missing and random enhancement procedures.

#### 2.1.2 Histopathological image data

The tissue microarrays (TMAs) of all eligible patients were constructed using tissue cores from formalin-fixed paraffin-embedded tissue of rectal cancer surgical resection specimens. Then, sections that were most representative of the depth of invasion in each case were selected by the Director of the Department of Pathology at The Affiliated Hospital of Xuzhou Medical University. Subsequently, all selected slides were scanned using the Aperio ScanScope Scanner system (Leica Biosystems) with the ×20 objective, and images were digitized as svs. format files. In light of the lower resolution of TMAs compared to other pathological images, we did not employ patch-based segmentation, but instead scaled the entire image to 2048×2048 pixels (one pixel is equal to 0.504 μm) and input it into the model. Considering that the limitation of the number of training samples can easily lead to model overfitting, we added data enhancement strategies including random luminance increase/decrease, rotation, flipping, mirroring, and random noise addition. The integrated probability of data enhancement is 70%, and 1-3 enhancement methods are randomly selected for each enhancement, in which the luminance increase or decrease is between 50% and 150%, and the random noise addition mainly includes Gaussian, Poisson, and pretzel noise.

### 2.2 Model algorithms and development

**Figure 1:**
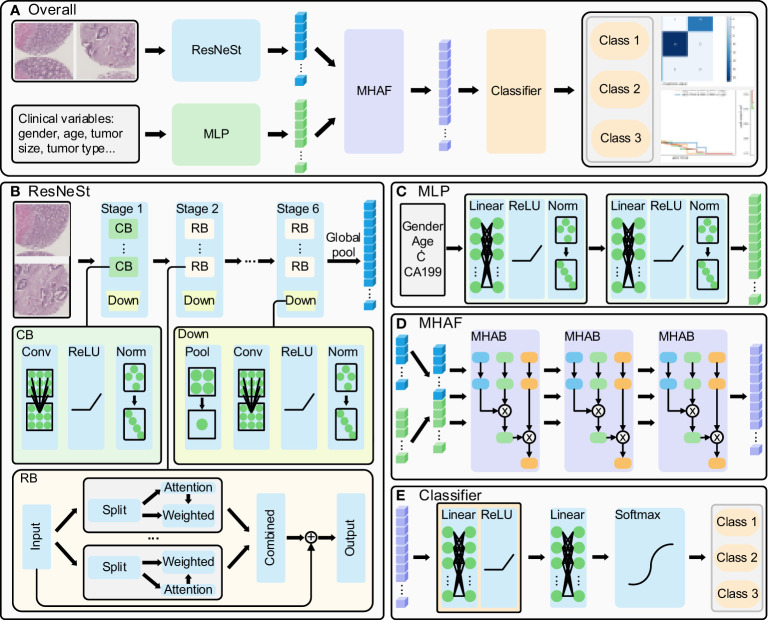
Multi-modal deep learning model. **(A)** overall flow of the designed method, **(B)** ResNeSt structure: extract the histopathological images features, **(C)** MLP structure: process the clinical parameters, **(D)** MHAF structure: fuse the image and clinical parameters and generate multi-modal features and **(E)** generate the rectal cancer survival prediction classification results.

The present study proposes a method whose overall flow is illustrated in **Figure 1A**. Firstly, ResNeSt is utilized to extract features from cancerous and paracancerous tissue microarray images, while clinical parameters are processed using a multi-layer perceptron (MLP); subsequently, multi-head attention fusion (MHAF) is applied to fuse the extracted image and clinical parameter features, thereby generating multimodal features; finally, the multimodal features are input into a classifier to obtain accurate classification results for rectal cancer prediction.

The ResNeSt model is a feature extraction model that has been adopted and improved from the ResNet model. It introduces the split-transform-merge and channel attention mechanisms to enhance its feature representation capability and propensity. The model’s structure is illustrated in **Figure 1B** (14, 15). The ResNeSt block is the core component of ResNeSt, which slices the input features, generates an attention vector based on the sliced features, weights the sliced features using the attention vector, and merges the weighted features by adding the residuals to output the attention features. Given the low resolution of tissue chip images, we have modified the number of ResNeSt downsampling while adjusting the number of convolution and layers in each stage of ResNeSt. The modified ResNeSt consists of six stages, where the first stage is mainly made up of a con-volution block, while the remaining five stages consist of the ResNeSt block. The first stage contains 2 convolution blocks, which have an output feature channel number of 8. Stages 2, 3, 4, 5, and 6 contain 3, 4, 5, 6, and 4 ResNeSt blocks, which have an output feature channel number of 16, 32, 64, 128, and 256, respectively. The number of training rounds for the network is 120, the learning rate has an initial value of 0.01 and decays to a minimum of 0.00001, and the optimizer uses AdamW. The convolution block includes the convolutional layer, activation function, and normalization layer. At the end of each stage, a downsampling is performed, primarily consisting of pooling, convolution, activation, and normalization. We first fed cancer tissue images and paracancer tissue images into the model for prediction separately.Then, we combine the cancerous and paracancerous 3-channel images to form a 6-channel matrix that is fed as input to ResNeSt. The six-channel matrix is globally pooled to generate the final image features after six downsampling stages.

The Multilayer Perceptron (MLP) is utilized to extract features from clinical parameters, and its structure is depicted in **Figure 1C**. It is comprised of two sets of fully connected blocks, each of which includes a linear layer, activation function, and normalization layer. The MLP employs weight manipulation and dimensionality expansion to generate clinical features that are congruent with image features.

After extracting features from images and clinical parameters, two sets of features are inputted into the Multi-Head Attention Fusion (MHAF) model with the structure depicted in **Figure 1D**. The two sets of features are merged into a combination of features and then iteratively passed through three Multi-Head Attention Blocks (MHABs). The MHAB process is represented by the following equation


$$Attention\left(Q,K,V\right)=softmax\left(\frac{QK}{\sqrt{d_{k}}}\right)V$$

$$head_{i}=Attention\left(QW_{i}^{Q},\;KW_{i}^{K},\;VW_{i}^{V}\right)$$

$$\begin{array}{c} Output=MultiHead\left(Q,\;K,\;V\right)\\ =Concat\left(head_{1},\;\ldots ,\;head_{H}\right)W^{O} \end{array}$$

where *Q*, *K*, and *V* are the three initial vectors of MHAB, *Q* and *K* together form the attention vector to weight *V* to generate attention features, where they are all equivalent to the input features, $d_{k}$ denotes the dimension of *K*, $W_{i}^{Q}$ , $W_{i}^{K}$ , and $W_{i}^{V}$ are the weights of *Q*, *K*, and *V* in the i head, respectively, and they are mainly implemented by the linear layer, *H* denotes the number of heads, and $W^{O}$ denotes the output weights. MHAF is more targeted to fuse image and clinical features to generate multi-modal features.

The architecture of the classifier for the terminal phase of the model is depicted in **Figure 1E**. The Classifier principally encompasses a series of fully-connected blocks, linear layers, and softmax functions. The fully-connected blocks include linear layers and activation functions. Upon inputting the fused features into the classifier, the two sets of linear modules carry out the ultimate integration of the features and provide scores for the respective categories, while the softmax function restricts category scores to be between 0 and 1, culminating in the output of patient survival categories.

### 2.3 Training strategies

To alleviate the challenges associated with model fitting, we employed a stepwise pre-training approach for each of the components. Specifically, we conducted separate pre-training processes for ResNeSt and MLP in conjunction with their respective classifiers, targeting image and clinical parameter classification. Once the pre-trained model parameters were obtained, we then loaded them and proceeded with the training of MHAF and its classifier, which were frozen after a single epoch. Lastly, we unfroze ResNeSt and MLP and proceeded with the training of the complete model.

For model training, we use cross-entropy loss as the loss function, which is described as:


$$L=-\frac{1}{C}\sum_{c=1}^{C}\left[y_{c}log{\hat{y}}_{c}+\left(1-y_{c}\right)log\left(1-{\hat{y}}_{c}\right)\right]$$

where *C* denotes the number of categories, $y_{c}$ denotes the true value of category c, and ${\hat{y}}_{c}$ denotes the predicted value. We use the cross-entropy loss function to calculate the loss values, chain derivatives based on the loss values, and the AdamW optimizer to update the model parameters.

### 2.4 Model evaluation

To evaluate the model performance, we plotted the confusion matrix and receiver operating characteristic (ROC) curve, and calculated the area under the curve (AUC) of ROC, while using the indicators of accuracy (ACC), sensitivity (SE), specificity (SP), positive predictive value (PPV), negative predictive value (NPV), and F1 score. (SE), specificity (SP), positive predictive value (PPV), negative predictive value (NPV), F1 score, etc., which can be expressed by the following equations:


$$ACC=\;\frac{TP+TN}{TP+FP+TN+FN}$$

$$SE\;=\;\frac{TP}{TP+FN}$$

$$SP\;=\;\frac{TN}{TN+FP}$$

$$PPV=\;\frac{TP}{TP+FP}$$

$$NPV=\;\frac{TN}{TN+FN}$$

$$F1\;score=\;\frac{2TP}{2TP+FP+\;FN}$$

where TP is the number of true positive samples, TN is the number of true negative samples, FP is the number of false positive samples and FN is the number of false negative samples.

## 3 Results

### 3.1 Study cohorts and dataset

**Figure 2:**
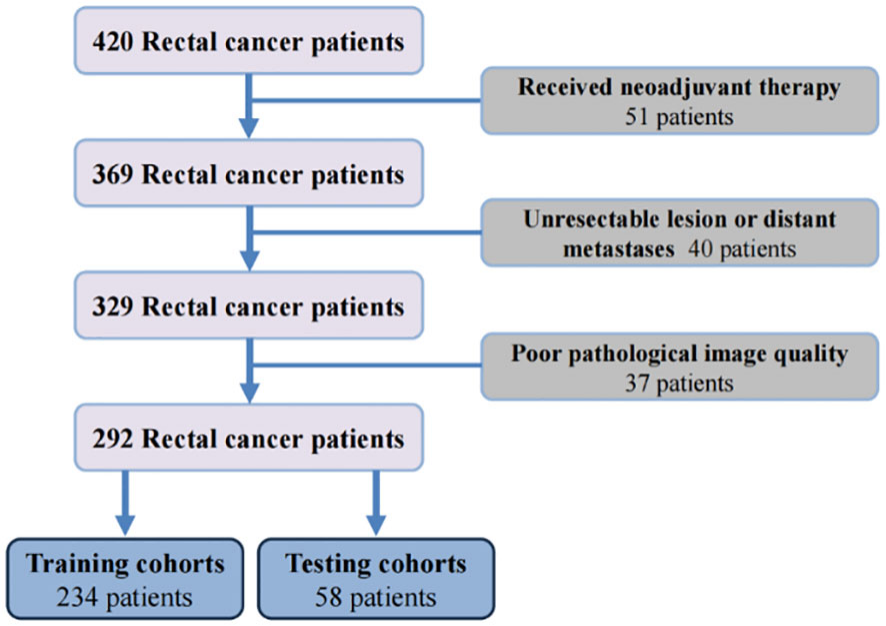
The flowchart of patients enrollment.

**Figure 3:**
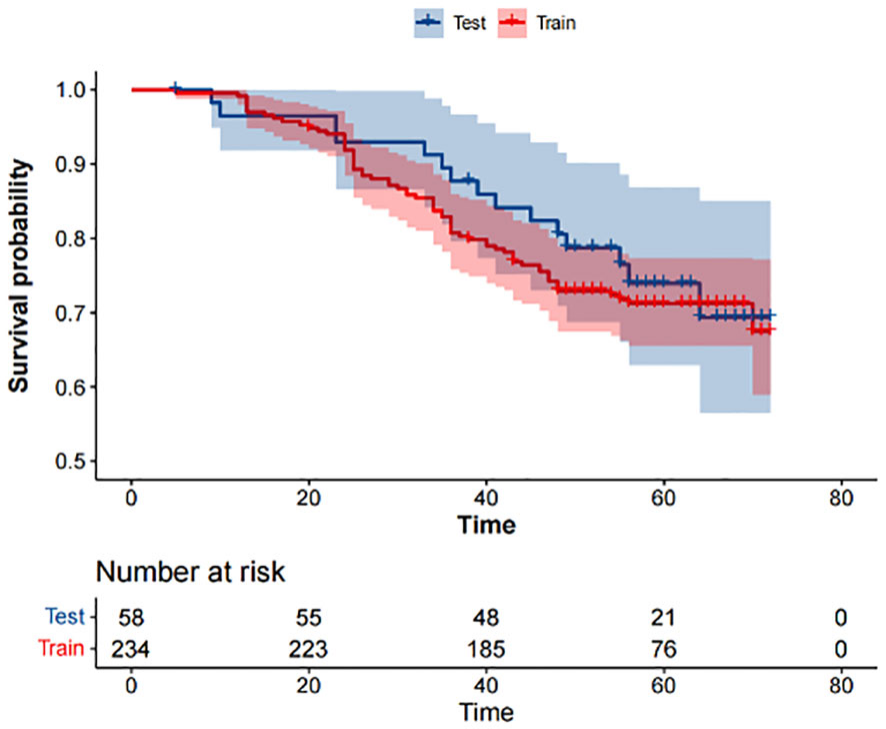
Kaplan-Meier survival analysis of the training and testing cohorts.

According to the inclusion and exclusion criteria, 292 patients were included in the study and were randomly allocated with a split ratio of 4:1 to the training and testing cohorts, respectively; 234 patients composed the training set, and 58 patients composed the testing cohort (**Figure 2**). **Table 1** lists the clinicopathological characteristics of patients in the training (n = 234) and testing (n = 58) cohorts. No significant difference in clinicopathological characteristics between the training and testing cohorts was found. In the training cohorts, 56.8% (133/234) were male, and the mean (SD) age was 63.6 (11.3) years. The majority of the patients (78.3%, 227/290) were diagnosed with stage II or III disease. The median follow-up duration (IQR) was 59 (57–61) and 59 (55–63) months in the training and testing cohorts, respectively. The K-M survival curves provide a more visual indication of the survival status of patients in the training and testing cohorts (**Figure 3**). The 5-year OS rate was 30.8% (72/234) in the training cohort. In the testing cohort, the 5-year OS rate was 34.5% (20/58). Based on the characteristics of the patients’ survival data collected in this study, we further stratified the patients’ OS into<4 years, 4-5 years, and more than 5 years.

**Table 1:** Characteristics of patients in the training and testing cohorts.

Characteristic	Training cohort(n=234)	Testing cohort (n = 58)	p-value
gender, No. (%)			0.99
female	101 (43.2)	25 (43.1)	
male	133 (56.8)	33 (56.9)	
age, mean ± Std	63.6 ± 11.3	63.9 ± 10.3	0.86
Tumor size, mean ± Std	4.2 ± 1.4	4.4 ± 1.6	0.43
Tumor type, No. (%)			0.60
0	6 (2.6)	0 (0.0)	
1	182 (77.8)	50 (86.2)	
2	46 (19.7)	8 (13.8)	
T, No. (%)			0.44
1	7 (3.0)	0 (0.0)	
2	60 (25.6)	15 (25.9)	
3	167 (71.4)	43 (74.1)	
N, No. (%)			0.77
0	144 (61.5)	36 (62.1)	
1	60 (25.6)	16 (27.6)	
2	30 (12.8)	6 (10.3)	
Grade, No. (%)			0.75
1	54 (23.1)	11 (19.0)	
2	90 (38.5)	25 (43.1)	
3	90 (38.5)	22 (37.9)	
ki67 (%), mean ± Std	31.2 ± 16.9	34.1 ± 18.9	0.25
P53, No. (%)			0.96
0	37 (15.8)	9 (15.5)	
1	197 (84.2)	49 (84.5)	
WBC, mean ± Std	6.5 ± 2.2	6.5 ± 2.0	0.94
RBC, mean ± Std	4.4 ± 0.5	4.4 ± 0.5	0.65
PLT, mean ± Std	230.8 ± 70.4	249.4 ± 71.7	0.08
HB, mean ± Std	130.7 ± 18.8	130.8 ± 13.9	0.97
LY, mean ± Std	1.8 ± 0.6	1.9 ± 0.6	0.69
AST, mean ± Std	20.4 ± 7.1	23.0 ± 22.6	0.14
ALT, mean ± Std	17.3 ± 9.1	20.5 ± 29.1	0.17
ALB, mean ± Std	42.9 ± 4.7	41.8 ± 4.4	0.10
Cr, mean ± Std	65.6 ± 15.5	67.8 ± 25.1	0.40
UREA, mean ± Std	8.9 ± 30.4	12.7 ± 36.8	0.43
CEA, mean ± Std	26.1 ± 108.2	27.1 ± 130.1	0.95
CA199, mean ± Std	36.9 ± 148.8	22.4 ± 42.2	0.46
OS(month), mean± Std	51.7 ± 15.6	53.3 ± 15.6	0.49
Survival interval (year), No. (%)			0.41
<4	70 (29.9)	14 (24.1)	
≥4, ≤5	92 (39.3)	24 (41.4)	
>5	72 (30.8)	20 (34.5)	

### 3.2 A deep learning framework for OS prediction from histopathological images

**Figure 4:**
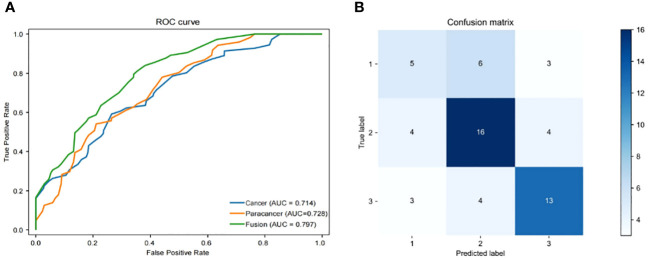
Performance of models. **(A)** Area under the ROC of H&E features (Cancer/Paracancer/Fusion), **(B)** Confusion matrix of H&E features (Cancer/Paracancer/Fusion).

The objective of this investigation was to develop a deep learning model to automatically predict 5-year overall survival (OS) in patients with rectal cancer utilizing H&E-stained tissue microarrays. We conducted a scan on H&E-stained tissue microarrays obtained from rectal cancer patients to acquire digital H&E-stained histopathological images. These images comprise H&E-stained histopathological images of both cancerous and paracancerous tissue for each patient. Paraneoplastic tissue refers to normal tissue located more than 3 centimeters from the tumor margin. We employed the ResNeSt model to analyze digital H&E-stained histopathological images of patients. Our aim was to utilize the predictive model, which we trained on cohorts, to forecast the overall survival of patients based on their H&E-stained histopathological images and to test the efficacy of the model in testing cohorts. Initially, we used cancer tissue images for prediction with an AUC of up to 0.714 (**Table 2**), alongside a specificity rate of 0.755. Subsequently, we used paracancerous tissue images for prediction purposes. The AUC of the model reached 0.724, with a specificity rate of 0.754. Lastly, we integrated the cancer tissue images together with the paracancerous tissue images of patients into the model for prediction. This achieved an AUC of 0.797, accompanying a specificity rate of 0.788. Combining two types of histopathological images into the model for training yields better prediction results than those predicted with only one type of histopathological images. **Figure 4A** depicts the ROC of the ResNeSt model with two different pathology images, whereas **Figure 4B** demonstrates its corresponding confusion matrix. From the confusion matrix, it can be seen that the model predicted correctly 5 of the 14 patients in the testing set whose actual survival was less than 4 years; the model predicted correctly 16 of the 24 patients whose actual survival was between 4 and 5 years; and the model predicted correctly 13 of the 20 patients whose actual survival was greater than 5 years, which can be seen that the model has a high prediction accuracy for patients whose actual survival is greater than 4 years, and that the prediction performance of the model is still to be improved for the patients with an actual survival period of less than 4 years.

**Table 2:** Comparison of model performance with different histopathological images.

	AUC	ACC	SE	SP	PPV	NPV	F1 score
Cancer	0.714	0.678	0.497	0.755	0.501	0.754	0.498
Paracancer	0.728	0.678	0.507	0.754	0.506	0.754	0.506
Fusion	0.797	0.724	0.558	0.788	0.561	0.79	0.558

### 3.3 Fusion of clinical features can improve the accuracy of the OS prediction model

**Figure 5:**
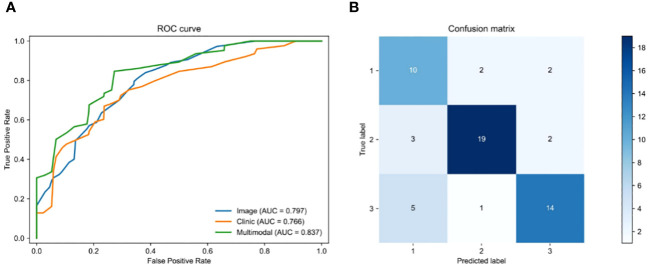
Performance of models. **(A)** Area under the ROC of H&E features combined with clinical features, **(B)** Confusion matrix of H&E features combined with clinical features.

We employed a multi-modal approach to integrate H&E image features with 21 clinical features in order to predict the survival status of rectal cancer patients. To ensure fairness in the comparison, we only fused clinical features and made no alterations to any other parameters of the model. The pre-processed histopathological images were fed into a ResNeSt deep learning model, while the clinical information was processed by an MLP deep learning model during training and testing. Once the features of the histopathological images and clinical parameters were extracted, they were fed into the MHAF model as two sets of features. These sets of features were then merged into hybrid features and input into three sets of MHAB models in sequence. Finally, classification was performed by a soft-max function to predict the survival status of rectal cancer patients. **Figure 5A** displays the ROC of the MHAF model with the fused image and clinical features, while **Figure 5B** demonstrates its corresponding confusion matrix.

We first entered the clinical parameters into the prediction model, which achieved an accuracy of 0.797 in the test set in the testing cohort. Secondly, we can see that the AUC of the model with combined clinical and image features was 0.837 (**Table 3**), which is 0.04 higher than the model with image features only. The accuracy was 0.828, which was an improvement of 0.104. The precision was 0.732, which was an improvement of 0.171. The recall was 0.735, which was an improvement of 0.177, and the F1 score was improved by 0.171. As shown by the confusion matrix, the model correctly predicted 10 of the 14 patients in the test set whose actual survival was less than 4 years, 19 of the 24 patients whose actual survival was between 4 and 5 years, and 14 of the 20 patients whose actual survival was greater than 5 years. The performance of the multimodal prediction model was significantly improved compared with the prediction results of the pathology image-based prediction model described above. The above results suggest that the fusion of image and clinical features is beneficial to improve the accuracy of predicting rectal cancer patient survival status.

**Table 3:** Comparison of the performance of three different models.

	AUC	ACC	SE	SP	PPV	NPV	F1 score
Images	0.797	0.724	0.558	0.788	0.561	0.79	0.558
Clinic	0.766	0.713	0.565	0.79	0.573	0.785	0.56
Multi-modal	0.837	0.828	0.735	0.875	0.732	0.87	0.729

## 4 Discussion

The task of accurately determining the survival of rectal cancer patients by the conventional TNM staging method has become increasingly difficult in recent times, as it no longer satisfies the accuracy standards set by contemporary medical practice. There is an urgent need for the development of novel techniques to accurately forecast the survival outcomes of individuals diagnosed with rectal cancer. In order to achieve this objective, we provide a multi-modal deep learning system for predicting survival, which combines H&E-stained histopathological pictures with clinical data. We have two pipelines in our job. The initial pipeline is the direct prediction of survival status using pre-processed rectal cancer tissue microarrays (TMAs). Our model employs ResNeSt algorithms to optimize the utilization of information contained in the histopathological images. The constructed prediction model incorporating pathological images of cancerous and paracancerous tissues had an AUC value of 0.797, an accuracy rate of 0.724 and a specificity rate of 0.788. The second pipeline utilizes an MHAF model to combine imaging data and clinical information in order to accurately forecast the survival status of rectal cancer patients. The results of the multi-modal prediction model were also as we had hoped. The AUC of our model achieved a commendable value of 0.837, accompanied with an accuracy rate of 0.828. The specificity rate was also improved to 0.875. The findings of our study suggest that the integration of histopathological pictures and clinical characteristics yields the most optimal outcomes in predicting survival rates for rectal cancer.

Our suggested strategy enhances the accuracy of predicting the survival status of rectal cancer patients by just utilizing clinical factors and histological pictures of patients. The clinical data collected for this study included general patient information, tumor tissue-related information, pathological staging, and laboratory test data. All of these data are related to the patient’s treatment process, and we analyzed these data may be related to the patient’s prognosis. Meanwhile, the advantage of our selected MLP algorithm is that it is able to acquire the information contained in a large amount of clinical data and construct complex models. Therefore, we incorporated these data into the multi-modal prediction model we constructed in the hope of improving the performance of the model as much as possible. The results are as expected, as shown by the confusion matrix, some patients’ survival is wrong in our prediction based on pathological image prediction model, and then correctly predicted in the multimodal prediction model with the addition of clinical data, which can reflect, on the other hand, the importance of the addition of clinical data to improve the performance of prediction models. This study also employed carcinogenic and paracancerous tissues from patients to estimate their life. It was shown that the paracancerous tissues had a strong ability to anticipate patient survival. These findings indicate that the tissues around the malignant area may possess significant tumor-related data that necessitates more investigation.

There are two computational methods for pathological images: traditional machine learning and deep learning (16, 17). Machine learning techniques are extensively employed in prognostic prediction to significantly decrease the time required for the diagnostic procedure (18–20). Convolutional neural network (CNN) is the most popular deep learning model for image processing at present (21–23). It may be utilized for both tumor identification and the quantitative analysis of cell properties in pathological images (24, 25), but also for the classification of small tissue images in pathological diagnosis (26, 27). The CNN algorithm is now considered the most advanced method for image identification and classification because to its consistent and reliable learning capabilities (28). The optimization of CNN may be achieved by employing the error backpropagation technique along with the gradient descent method. Nevertheless, once a specific depth is achieved, augmenting the number of layers in the Convolutional Neural Network (CNN) no longer yields any enhancements in the classification accuracy. The problem is addressed with ResNet. ResNeSt is an enhanced version of ResNet that incorporates a Split-Attention module into the architecture design, resulting in increased performance of the network (14, 15). The Multi-Layer Perceptron (MLP) is a type of feedforward ANNs (Artificial Neural Network) that, which has a long history of implementation in medical research for image classification (29, 30), detection (31, 32) and prediction (33, 34). The multi-head attention mechanism aims to prioritize the most relevant information for the current task from a large amount of input data. By reducing attention to other information, it addresses the issue of information overload and enhances the efficiency and accuracy of task processing. Overall, we have selected several algorithmic models to handle the data depending on distinct data categories. This selection is aimed at achieving accurate predictions.

Oncology is now experiencing a growing utilization of multi-modal deep learning. Huang et al. introduced a multi-modal deep learning model that utilizes a ResNet and multimodal compact bilinear pooling technique to directly predict the tumor mutational burden (TMB) status from histopathological images and clinical data. The AUC of the model with combined clinical and image features was 0.817, which is 0.013 higher than the model with image features only. The accuracy was 0.872, which was an improvement of 0.022. The precision was 0.748, which was an improvement of 0.027 (35). Qiu et al. initially investigated the correlation between MSI status and several molecules, such as mRNA, miRNA, lncRNA, and DNA methylation (30). Subsequently, a new and innovative deep learning framework was created to accurately forecast MSI status using just hematoxylin and eosin (H&E) staining pictures. This was achieved by integrating the H&E image with the aforementioned molecules by multimodal compact bilinear pooling. The findings shown that the fusion models that combine H&E images with a single kind of molecule have superior prediction accuracies compared to those that just utilize H&E images. Hao et al. developed and assessed a complex neural network architecture called SurvivalCNN for the purpose of predicting the survival of patients with stomach cancer. The model incorporates several threads and modalities to enhance its predictive capabilities (36). Using five-fold cross validation, the experimental findings demonstrate that SurvivalCNN obtains an average concordance index of 0.849 for predicting OS and 0.783 for predicting PFS. Wang et al. introduced a multi-modal deep learning radiomics method to forecast the immunotherapy response of gastric cancer patients. Firstly, they trained a random forest classifier based upon the radiomics features only, which achieved an AUC of 0.677 and 0.728 in the internal and external validation cohorts, accordingly. Then, they utilized both clinical data and computed tomography images, resulting in an AUC of 0.812 (37). Nevertheless, there is a lack of studies that employ multi-modal deep learning techniques to forecast overall survival in individuals diagnosed with rectal cancer. The multi-modal deep learning model we developed shown strong predictive capability for overall survival (OS) in patients with rectal cancer. Once the suggested model is validated in the future, it might be used as a clinical tool to enhance the calculation of survival and study of prognosis for patients with rectal cancer.

Presently, our approach is subject to some constraints. Initially, it is worth noting that our model exhibited high values for both the AUC (0.837) and specificity (0.875). However, the evaluation metrics, namely precision, recall, and F1 score, were comparatively low. This suggests that there is room for improvement in terms of the accuracy and sensitivity of our model. Enhancing the prediction performance might potentially be achieved by implementing more sophisticated classification techniques (10, 38). Furthermore, this study exclusively used clinical data. Integrating picture characteristics with multi-omics data and other factors has the potential to enhance the accuracy of predicting survival outcomes (20, 39). Ultimately, we failed to verify our model using other data, potentially affecting the correctness of our model. The methodology we have suggested needs ongoing optimization and testing in future clinical practice.

## 5 Conclusion

In summary, we designed and evaluated a deep learning based multi-modal structure for rectal cancer patient survival prediction. Our experiments on a large clinical dataset demonstrated the superior effects of the proposed methods. With future validation, the proposed model may serve as a software tool to improve clinical patient survival estimation and prognosis analysis.

## Data availability statement

The data are not publicly available due to privacy or ethical restrictions. Requests to access the datasets should be directed to songjun@xzhmu.edu.cn.

## Ethics statement

The studies involving humans were approved by The Affiliated Hospital of Xuzhou Medical University Medical Ethics Committee. The studies were conducted in accordance with the local legislation and institutional requirements. The participants provided their written informed consent to participate in this study. Written informed consent was obtained from the individual(s) for the publication of any potentially identifiable images or data included in this article.

## Author contributions

YX: Writing – review & editing. JG: Writing – original draft. NY: Writing – original draft, Investigation. CZ: Writing – original draft, Data curation. TZ: Writing – review & editing, Project administration. WZ: Writing – review & editing, Software. JL: Writing – review & editing, Supervision. JS: Writing – review & editing, Funding acquisition.
